# Mechanics of near-field deformation during co- and post-seismic shallow fault slip

**DOI:** 10.1038/s41598-020-61400-9

**Published:** 2020-03-19

**Authors:** Johanna M. Nevitt, Benjamin A. Brooks, Rufus D. Catchings, Mark R. Goldman, Todd L. Ericksen, Craig L. Glennie

**Affiliations:** 1 U.S. Geological Survey, P.O. Box 158, Moffett Field, CA 94035 USA; 2 Department of Civil and Environmental Engineering, University of Houston, Houston, TX 77204 USA

**Keywords:** Natural hazards, Structural geology, Seismology

## Abstract

Poor knowledge of how faults slip and distribute deformation in the shallow crust hinders efforts to mitigate hazards where faults increasingly intersect with the expanding global population at Earth’s surface. Here we analyze two study sites along the 2014 **M** 6.0 South Napa, California, earthquake rupture, each dominated by either co- or post-seismic shallow fault slip. We combine mobile laser scanning (MLS), active-source seismic tomography, and finite element modeling to investigate how deformation rate and mechanical properties of the shallow crust affect fault behavior. Despite four orders-of-magnitude difference in the rupture velocities, MLS-derived shear strain fields are remarkably similar at the two sites and suggest deceleration of the co-seismic rupture near Earth’s surface. Constrained by the MLS and seismic data, finite element models indicate shallow faulting is more sensitive to lithologic layering and plastic yielding than to the presence of fault compliant zones (i.e., regions surrounding faults with reduced stiffness). Although both elastic and elastoplastic models can reproduce the observed surface displacement fields within the uncertainty of MLS data, elastoplastic models likely provide the most reliable representations of subsurface fault behavior, as they produce geologically reasonable stress states and are consistent with field, geodetic, and seismological observations.

## Introduction

Investigators of the 1906 **M** 7.9 San Francisco earthquake produced what remains one of the most rigorous characterizations of fault rupture at Earth’s surface, including detailed records of off-fault plastic deformation^1^ and inferred shallow slip gradients^2^, phenomena that continue to puzzle earth scientists today. Since these pioneering studies, process-based knowledge of surface rupture and shallow deformation has remained elusive, due largely to the paucity of spatially coherent geodetic data close to active faults. This data gap is further compounded by the poorly understood mechanical behavior of the shallowest portion of Earth’s crust, where deeper, competent bedrock typically transitions into poorly consolidated, chemically-altered units and/or alluvium. For example, attempts to explain the hypothesized “shallow slip deficit,” an apparent discrepancy between fault slip in the shallow (0–3 km) and deeper (>~3 km) crust^3,4^, have appealed to both improved data coverage^5,6^ and better quantified uncertainty in the constitutive properties of the shallow crust^6–8^. Importantly, our lack of knowledge regarding shallow fault behavior impedes our ability to forecast seismic hazard, with significant financial and public safety consequences^9^. For instance, the Uniform California Earthquake Rupture Forecast^10^ and U.S. Geological Survey National Seismic Hazard Maps^11^, which inform insurance rates and emergency plans, depend on measurements of fault slip at Earth’s surface that do not consider possible subsurface variations^2,12^. Additionally, the emerging field of Fault Displacement Hazard Analysis, which aims to improve construction practices near faults (e.g., California’s Alquist-Priolo Act^13^), relies on empirical relations that neglect variations in the mechanical properties of the host lithology^14^.

Advances in near-field geodetic techniques allow us to accurately quantify displacement fields near faults with unprecedented spatial resolution, often revealing distributed (or “off-fault”) deformation that is more complex than originally assumed^12,15–19^. Two factors receiving significant attention for their potential to affect fault slip and distributed deformation are compliant zones^6,20,21^ and elastoplasticity^7,8,22–24^. Compliant zones correspond to densely fractured regions surrounding faults with reduced elastic stiffness^25^, particularly following earthquakes^26,27^, that locally reduce seismic velocities^28,29^ and enhance elastic deformation^21^. Previous studies have argued, however, that distributed deformation around some faults cannot be fully accounted for by elastic strain within compliant zones^30,31^. Such deformation may arise from mechanisms of plastic yielding, for which evidence exists in paleoseismic trenches^32,33^. Although gravitational effects in layered media, including the acceleration of viscoelastic relaxation at long wavelengths and attenuation of the overall vertical displacement field, become important at length scales greater than several elastic plate thicknesses and/or over time periods much greater than the relaxation time, the effect on strains is negligible in the near-field and within a single earthquake cycle^34–38^.

Relatively unstudied factors that may affect near-field deformation include layered elastic properties^39,40^ and deformation rate (e.g., co-seismic versus post-seismic slip)^41,42^. Without greater confidence in how these proposed factors affect faulting near Earth’s surface, we cannot reliably use the results of near-field geodetic analyses to infer shallow deformation, nor can we formulate models to predict fault slip and deformation in future events.

## A high-resolution comparison of co- and post-seismic deformation

**Figure 1:**
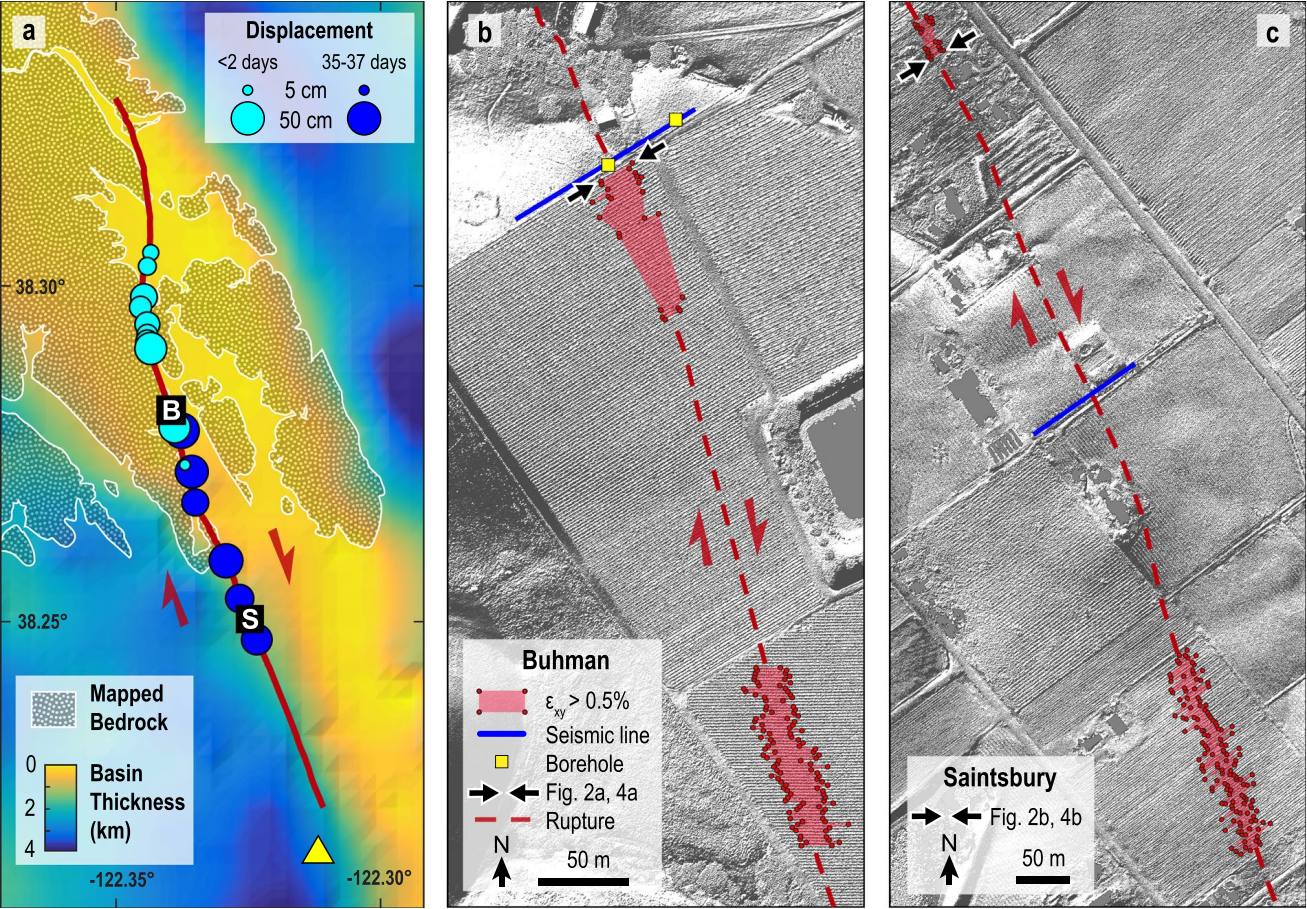
Overview of the 2014 **M** 6.0 South Napa earthquake rupture and study sites. (**a**) The earthquake rupture nucleated in the south (yellow triangle) and propagated along the principal rupture (red) of the West Napa Fault Zone (WNFZ) northward across a prominent change in surface geology and basin thickness inferred from gravity measurements^12,111^. Regions that are not stippled represent Quaternary alluvium and sediments^62^. Cyan circles are field measurements of predominantly co-seismic offsets made <2 days following earthquake. Blue circles are predominantly post-seismic shallow slip inferred from MLS measurements made 35–37 days following the earthquake. Buhman (38.281088, −122.339080) and Saintsbury (38.250020, −122.324250) study site locations are indicated by “B” and “S,” respectively. (**b,c**) Site details for Buhman and Saintsbury, respectively, showing rupture trace, vine rows analyzed, and seismic survey locations. Red shading indicates regions with high shear strain (ε_xy_ > 0.5%), likely exceeding the elastic limit. Base maps are digital elevation models generated using LasTools and GMT software from the 2014 South Napa Earthquake Airborne Lidar dataset hosted by OpenTopography^48,112^.

The **M** 6.0 South Napa, California, earthquake rupture (2014-08-24 10:20:44 UTC) nucleated at a depth of 9.4 km and propagated unilaterally updip along the West Napa Fault Zone (WNFZ), approximately following the base of a northward-shallowing alluvial basin^43–47^ (Fig. 1a). Deformation at Earth’s surface occurred along several strands of the WNFZ, spanning ~2.5 km in width and ~12 km in length^48^. Minor co-seismic surface offsets (<5 cm) were observed on the eastern subsidiary fault strands^43^. We focus on the westernmost strand, which produced the majority of co-seismic and all post-seismic surface deformation^12,47,49,50^. On this principal strand, co-seismic surface deformation was largely confined to the north where the shallow rupture passed through sedimentary bedrock of the Great Valley Group^51^. Within the basin to the south, surface deformation primarily accrued post-seismically, eventually matching the northern co-seismic surface offsets (Fig. 1a)^12,50,52^. Thus, the South Napa earthquake rupture provides a unique opportunity to study deformation at Earth’s surface arising from either co-seismic or post-seismic slip under otherwise similar conditions.

Hundreds of fault-crossing grape vines recorded surface deformation due to the South Napa earthquake in high-resolution. A deformed “vine row” typically displays continuous right-lateral deflection across a 5 to 30 m wide zone containing a narrower array of left-stepping, echelon, mixed-mode fractures^12^. Although paleoseismic studies suggest that the WNFZ previously ruptured to within 2–3 m of Earth’s surface, trench excavations and inversions assuming elastic deformation of the vine rows indicate the primary rupture during the 2014 South Napa event remained buried ≥3 m, regardless of whether near-surface faulting was co- or post-seismic^12^. Additionally, continuous drill core collected at a site that experienced post-seismic surface deformation contains a localized (2–3 cm wide) clay-rich shear zone at a depth of 9 m^53^, indicating the primary rupture terminated between ~3–9 m depth at that location. Our conclusion that the rupture remained buried does not preclude the occurrence of distributed deformation off the principal fault surface. In fact, preliminary analysis of the continuous drill core reveals off-fault subsidiary structures with individual offsets <1 cm^53^. Thus, the South Napa earthquake rupture occurred on a pre-existing fault plane, but did not breach Earth’s surface co- or post-seismically.

We target two sites along the principal fault rupture, Buhman (Fig. 1b) and Saintsbury (Fig. 1c), that express surface deformation dominated by co- or post-seismic shallow slip, respectively. At Buhman, the co-seismic rupture likely reached the near-surface within ~5 s^45,46^, suggesting an average hypocenter-to-site rupture velocity of 2.7 km/s. Landowners first surveyed the site ~4 hours post-earthquake, finding two fault-crossing PVC pipes had broken^54^. The first scientific measurement, made 35 hours post-earthquake, recorded a single vine row offset 40–45 cm^51^. Subsequent lidar surveys of 80 vine rows, made 9 and 37 days post-earthquake, found average offsets to be 45 ± 8 cm and 44 ± 8 cm, respectively, suggesting that any post-seismic deformation was not significant^12^. In contrast, surface deformation at Saintsbury was predominantly post-seismic. A fault-crossing road ~500 m northwest of Saintsbury was undeformed ~3 hours post-earthquake, where surveyors found a 10 cm high scarp the following day^51^. Kinematic finite fault models show the top of the co-seismic rupture at approximately 2 km^47^ to 2.5 km^45,46^ depth at Saintsbury. Taking 3 hours as the minimum time for slip to reach the near-surface, the estimated post-seismic rupture velocity from the co-seismic slip patch to the surface was 1.9 × 10^−4^ to 2.3 × 10^−4^ km/s, four orders-of-magnitude slower than at Buhman. Alignment arrays, repeat laser scans, and radar interferometry all indicate substantial afterslip near Saintsbury, producing 40–50 cm total surface offset ^12,50,52,55^.

**Figure 2:**
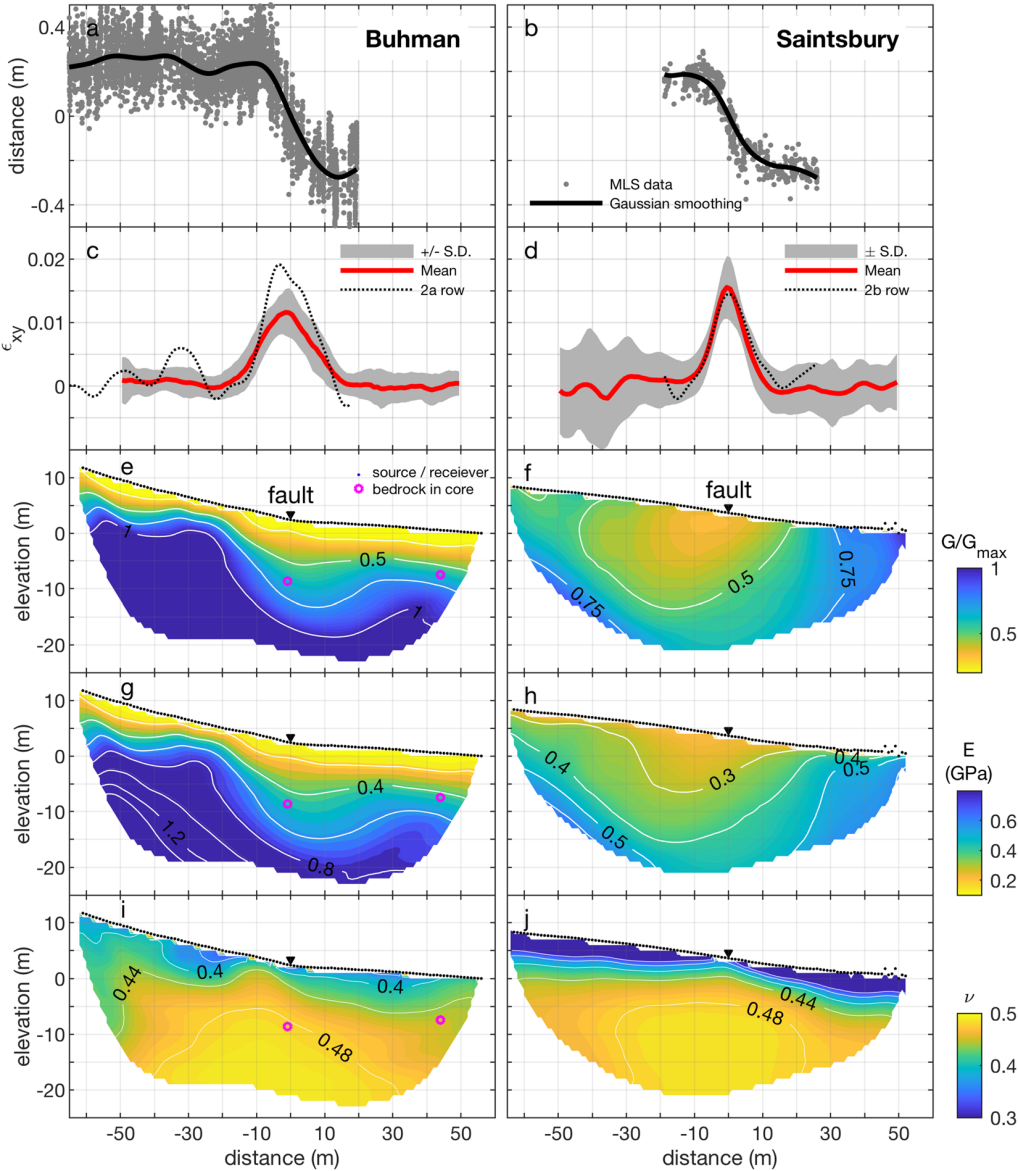
Surface deformation and elastic properties for the Buhman (left) and Saintsbury (right) sites. (**a,b**) MLS imaging in map-view of example vine rows from each site, with non-parametric Gaussian smoothing function; (**c,d**) Shear strain profiles averaged for 69 vine rows at Buhman and 83 vine rows near Saintsbury; (**e,f**) Shear modulus normalized by the maximum value (250 MPa) at Saintsbury; (**g,h**) Young’s modulus; (**i,j**) Poisson’s ratio. Tomography is vertically exaggerated 1.6 times and is shown only where there are ≥2 ray paths. Distance along the abscissa is measured from fault rupture trace.

Adjacent to each study site, we used vehicle-mounted mobile laser scanning (MLS) to image vineyard deformation within ±~50 m of the fault. The MLS point cloud for a typical vine row includes >50,000 laser returns, each referenced in three dimensions at approximately centimeter-scale resolution and accuracy^12^, which we smooth using non-parametric Gaussian kernel regression in order to calculate shear strain (Methods, Fig. 2a,b). The averaged shear strain profiles for all analyzed vine rows (Fig. 2c,d) indicate similar deformation at the two sites, with a slightly wider shear zone (~35 m) and less shear strain (peak average = 0.012) at Buhman compared to Saintsbury (~25 m and 0.015, respectively). Notably, shear strain at both sites exceeds the expected limit for elastic strain (~0.5%^12,56^) (Fig. 1b,c), suggesting plastic yielding occurred. This is consistent with penetrative shear fabrics observed in trenches at both sites^12^ and the lower-than-expected ground-motions at intermediate to far distances during the South Napa earthquake^57^, which may suggest near-fault plastic deformation^58–60^.

## Elastic structure underlying Earth’s surface deformation

We use high-resolution (1 m source/receiver spacing) active-source seismic surveys to infer the subsurface seismic velocity structure and corresponding distribution of elastic properties at the Buhman and Saintsbury sites^61^. Details on seismic data acquisition and processing to constrain elastic moduli are presented in the Methods section. We calculate elastic moduli assuming uniform density and isotropic linear elasticity. The assumption of uniform density is the primary source of uncertainty in the elastic moduli, which we discuss in Supplementary Note 1 and illustrate in Supplementary Figs. 1–6. In contrast to the similar surface deformation observed at the two sites (Fig. 2a–d), tomographic models indicate starkly different magnitudes and distributions of elastic moduli (Fig. 2e–j).

At Buhman, the seismic data indicate that the shear (*G*) and Young’s (*E*) moduli vary significantly with depth, with a soft layer (*G* ≈ 60 MPa, *E* ≈ 200 MPa) running sub-parallel to the surface topography, and a stiff layer (*G* ≤ 625 MPa, *E* ≤ 2 GPa) forming an asymmetric step at depth (Fig. 2e,g). Supported by preliminary drilling results (Supplementary Note 2, Supplementary Fig. 7), we interpret that the *E* = 0.5 GPa contour approximately corresponds to a transition between poorly consolidated sediments and Great Valley Group sandstone^62^. Poisson’s ratio (*ν*) varies from ~0.4–0.49 (Fig. 2i). The *ν* = 0.44 contour, which likely represents the water table^61^, occurs at ~5 m depth below the rupture trace. Poisson’s ratio is very high (ν > 0.48) for material surrounding the fault within +30/−40 m horizontal distance. Because ν = 0.5 defines an incompressible elastic material, we interpret that this near-fault zone is fluid-saturated^63,64^. Elevated Poisson’s ratio also is consistent with damage (i.e., fractures) surrounding the fault^65^.

At Saintsbury, the seismic data suggest *G* and *E* vary smoothly in a bowl-shaped distribution slightly off-centered from the rupture trace (Fig. 2f,h), in contrast to the subhorizontal layering at Buhman. The Saintsbury distribution, with soft material (*G* ≈ 85 MPa, *E* ≈ 300 MPa) in the center grading outward to stiffer material (*G* ≈ 250 MPa, *E* ≈ 600 MPa), agrees with previous characterizations of fault compliant zones^28,61^. Relative to maximum *G* in this survey, the compliant zone representing a 50% decrease in rigidity is ~50 m wide. As at Buhman, the *ν* = 0.44 contour occurs at ~5 m depth below the rupture trace and a highly incompressible region (ν > 0.48) surrounds the fault within +20/−30 m horizontal distance.

Comparing the seismic tomography models with the MLS data (Fig. 2) reveals a surprising insensitivity of surface deformation to variations in subsurface elastic properties. Buhman displays prominent horizontal layering of a soft surface unit overlying stiffer basement, whereas Saintsbury is characterized by a well-defined compliant zone surrounding the fault. The corresponding surface deformation fields, however, do not obviously respond to these distinct elastic structures. In addition, the compliant zone width at Saintsbury (Fig. 2f) is nearly twice that of the observed shear zone (Fig. 2d), further indicating that the compliant zone does not strictly control the strain distribution. Our estimate of compliant zone width represents a minimum constraint^28^, since surveys covering the greater Napa region found a >400 m wide compliant zone with a 35–50% relative velocity reduction, and a more prevalent ~200 m wide low-velocity zone at shallow depths^66,67^, providing an even poorer match to the MLS data.

## Sensitivity of shallow fault behavior to variations in mechanical properties

**Figure 3:**
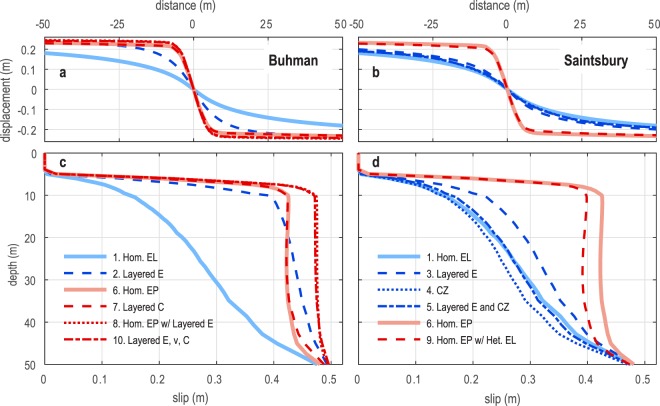
Modeled surface displacement (**a,b**) and subsurface slip (**c,d**), testing the range of mechanical properties characteristic of the Buhman and Saintsbury sites for a fault buried 5 m below Earth’s surface. Elastic models are shown in blue, elastoplastic models in red. Homogeneous elastic and elastoplastic model results provide points of reference for comparison across both Buhman (**a,c**) and Saintsbury (**b,d**) based models. Mechanical properties for each model are reported in Table 1. Below 50 m, fault slip is uniform and prescribed to be 0.5 m.

Mechanical models allow us to test systematically how variations in mechanical properties affect fault behavior. We do this using 3D quasi-static finite element models and the range of mechanical properties inferred using seismic tomography for the Buhman and Saintsbury study sites (Fig. 3, Table 1). Details on model development, implementation, and benchmarking are included in the Methods section and Supplementary Information.

**Table 1:** Mechanical properties used in the finite element models.

Model Name^a^ (Fig. 3)	E_b_^b^ (MPa)	E_b_^cz^ (MPa)	E_s_ (MPa)	E_s_^cz^ (MPa)	ν_b_	ν_b_^cz^	ν_s_	ν_s_^cz^	C_b_ (kPa)	C_s_ (kPa)	ϕ (°)	Ψ (°)
1. Hom. EL *(both sites)*	600				0.44				—			
2. Layered E *(Buhman)*	2000		200		0.44				—			
3. Layered E *(Saintsbury)*	600		300		0.44				—			
4. CZ *(Saintsbury)*	600	300	600	300	0.44	0.48	0.44	0.48	—			
5. Layered E and CZ *(Saintsbury)*	600	400	400	300	0.44	0.48	0.3		—			
6. Hom. EP *(both sites)*	600				0.44				50		25	15
7. Layered C *(Buhman)*	600				0.44				15000	50	25	15
8. Hom. EP w/ Layered E *(Buhman)*	2000		200		0.44		0.4		50		25	15
9. Hom. EP w/ Het. EL *(Saintsbury)*	600	400	400	300	0.44	0.48	0.3		50		25	15
10. Layered E, ν, C *(Buhman)*	2000		200		0.44		0.4		15000	50	25	5

^a^In the model names, “Hom.” indicates a homogeneous model, “EL” indicates a linear elastic model, “EP” indicates an elastoplastic model, “CZ” indicates a model with a compliant zone, “Layered” indicates a model with lithologic layering of the identified parameters, “Het.” indicates a model with both a compliant zone and lithologic layering.

^b^E is Young’s modulus, ν is Poisson’s ratio, C is cohesion, ϕ is the angle of internal friction, and Ψ is the dilation angle. Subscripts “b” and “s” and superscript “cz” designate basement unit, surface unit, and compliant zone, respectively (Supplementary Fig. 2).

Each model includes a vertical, planar fault buried 5 m below the (Earth’s) free surface (Supplementary Fig. 8), consistent with previous estimates for the South Napa earthquake rupture^12^. Our analysis focuses on near-field deformation within ±50 m of the fault, which is not significantly affected by variable fault slip below 50 m depth (Supplementary Fig. 9, Supplementary Table 1). Consistent with observed far-field (>50 m from the South Napa rupture) surface displacements of ~0.5 m^12^, the models prescribe 0.5 m uniform slip along the fault at depths ≥50 m to drive shallower deformation. At depths ≤50 m, models solve for fault slip using the Coulomb criterion^68^ with *μ* = 0.4, based on laboratory testing of WNFZ material^12^. The model continuum is partitioned horizontally at z = −10 m and vertically at x = ±25 m to introduce mechanical heterogeneities representative of the prominent lithologic layering at Buhman and the compliant zone at Saintsbury (Methods, Supplementary Fig. 8). Motivated by the geologic (i.e. trench observations^12^), geodetic (i.e., strain fields in Figs. 1 and 2), and seismological evidence (i.e., lower-than-expected ground motions^57,58^) for plastic yielding during the South Napa event, we define the continuum constitutive behavior as either linear elastic or Mohr-Coulomb elastoplastic. Frictional plastic yield criteria, including Mohr-Coulomb and Drucker-Prager formulations, were originally developed to simulate the failure of granular materials^69^, and commonly are used to model fault-driven deformation in the shallow crust^7,70^ (see Methods for additional yield criteria details). Elastic properties are based on the seismic tomography models (Fig. 2) and plastic parameters assume previously published values for a cohesive soil^71^ and sandstone^72^ (Table 1).

The suite of models (Table 1) is divided into two classes, each broadly accounting for the mechanical property variations at the two study sites. The homogeneous elastic model results in shallow slip decreasing monotonically toward Earth’s surface where deformation is distributed across a broad zone (>±50 m from the fault) (Fig. 3). Whereas varying ν has little effect (Supplementary Fig. 10), varying *E* can significantly alter the resulting deformation. For the Buhman-based model, where layered surface and basement units have *E* = 200 MPa and 2 GPa, respectively, the pronounced stiffness contrast facilitates ~75% greater slip compared to the homogeneous elastic model at 20 m depth, and surface deformation largely focuses within ±25 m from the fault (Fig. 3a,c). The effect is less pronounced for the Saintsbury-based model, where the surface and basement layers have *E* = 300 MPa and 600 MPa, respectively, and fault slip is only ~25% greater compared to the homogeneous elastic model at 20 m depth (Fig. 3d). Introducing a compliant zone (Table 1) to the Saintsbury-based model results in only a ~10% reduction in slip compared to the homogeneous elastic model at 30 m depth, with slightly greater surface displacements. However, the simultaneous effects of the horizontal layers and compliant zone counteract each other, such that the resulting deformation is very similar to the homogeneous elastic model result (Fig. 3b,d).

Introducing elastoplastic properties results in markedly different slip behavior, with more slip reaching the near-surface and greater localization of surface deformation compared to elastic models (Fig. 3). This effect is due to plastic yielding around the fault tip, which enhances fault slip^73^, rather than yielding adjacent to the fault, which would dampen slip^7,8^. Note that this result is specific to the case of a buried, non-propagating fault tip and different behavior would be expected for a surface-rupturing fault. Incorporating realistically heterogeneous plastic properties has a smaller effect on the deformation than does varying elastic properties. For instance, the slip distribution resulting from the Buhman-based model with layered cohesion is nearly identical to the homogeneous elastoplastic model, while the model with homogeneous plastic properties and layered elastic properties results in ~10% greater slip at 10 m depth (Fig. 3c). This likely is because the heterogeneous elastoplastic model defines cohesion in the surface unit with the value assumed throughout the homogeneous model (Table 1). In both models, however, plastic yielding localizes within the surface unit around the fault tip, so the increased basement cohesion of the heterogeneous model has little effect.

## Inferring subsurface fault behavior from constrained forward models

**Figure 4:**
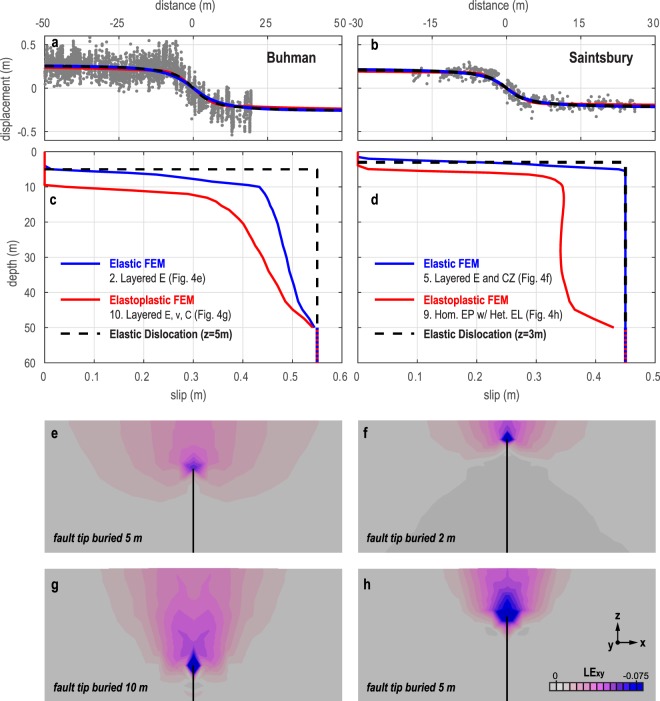
Results of forward finite element models (FEMs) with site-specific mechanical properties for the Buhman (left) and Saintsbury (right) sites. (**a,b**) Comparison of modeled surface displacements and MLS data (grey dots); (**c,d**) Modeled slip versus depth. Slip is prescribed (0.55 m for Buhman, 0.45 m for Saintsbury) below 50 m depth for all FEMs aside from Saintsbury Elastic FEM, which requires prescription to 5 m depth in order to fit MLS data; (**e**–**h**) Shown in cross-section, the distribution of logarithmic shear strain around the buried fault tip for each of the models in (**c,d**). Average misfit of modeled surface displacements from smoothed data (Fig. 2a,b) is nearly equal for elastic and elastoplastic model results: 4.0 and 3.4 cm, respectively, at Buhman, and 3.4 and 3.8 cm, respectively at Saintsbury.

Founded on the MLS data and seismic tomography models for the Buhman and Saintsbury sites, we develop geodetically- and mechanically-constrained forward models of shallow faulting (Fig. 4). Fitting the MLS data at each site requires different fault burial depths for elastic and elastoplastic models (Fig. 4a–d), with shallower depths at Saintsbury (2 m and 5 m, respectively) compared to Buhman (5 m and 10 m, respectively). As a result, models producing near-identical surface displacements at each site generate distinct subsurface slip distributions (Fig. 4c,d) and strain fields (Fig. 4e–h). Matching an elastic model to the Saintsbury MLS data requires that we prescribe slip to a shallower depth (5 m) than in the other models (50 m). Without this shallower prescription, slip tapers off at 50 m depth (Fig. 3d), and the modeled surface displacements poorly match the MLS data. This suggests that either a uniform slip front reached ~5 m depth, or, more likely, an elastic constitutive law does not accurately characterize deformation at the Saintsbury study site.

Common to each model (Fig. 4) is an abrupt decrease in slip near the fault tip (Fig. 4c,d). This high slip gradient is needed to fit the relatively straight limbs of the vine rows past ±~15 m from the fault, and can be produced by models with strongly contrasting elastic layers (e.g., Buhman) and/or plastic yielding near the fault tip. Models that include plastic yielding are more consistent with the previously discussed field observations. In addition, we prefer the elastoplastic models because they do not invoke unrealistically high stresses near the buried fault tip (Mises equivalent stress <~500 kPa), whereas the elastic models do (Mises equivalent stress >10 MPa). Furthermore, the elastic model for Saintsbury requires a fault tip burial depth of only 2 m, which is inconsistent with trench observations near that study site^12^. Although the models do not determine the cause of shallow rupture termination, models allowing the fault to reach Earth’s surface do not match the MLS data (Supplementary Fig. 11).

## Discussion

This study provides the first direct comparison of surface deformation resulting from dominantly co- versus post-seismic shallow fault slip. Despite four orders-of-magnitude difference in rupture velocity, surface deformation fields at the two study sites are surprisingly similar. We would expect that rapid co-seismic slip at Buhman would be accompanied by a transient dynamic stress field^74^ that would leave a permanent signature due to plastic yielding (Fig. 1b). In contrast, we would expect the slowly applied load during post-seismic slip at Saintsbury to be approximately balanced in static equilibrium with no dynamic plastic yielding. One explanation for the similar deformation fields at the two sites (Fig. 2a–d) is shallow deceleration of the co-seismic rupture at Buhman. Because inertial forces become insignificant at rupture velocities <~60% of the shear wave velocity^41,74^, the decelerating rupture may have allowed shallow deformation to accrue quasi-statically, thus approximating the dominantly post-seismic Saintsbury site. The hypothesis of rupture deceleration at Buhman is consistent with the buried rupture tip at ~10 m depth (Fig. 4g), and inversions indicating slower slip with delayed onset at depths <3–4 km near that site^45^. Thus, deformation observed at Earth’s surface may not be a reliable indicator of seismic versus aseismic shallow fault slip if ruptures decelerate in near-surface materials.

This study also highlights the outstanding question of why the principal South Napa earthquake rupture terminated just meters below Earth’s surface after traversing kilometers up-dip through the crust^12^. This behavior is not unique to the South Napa earthquake. For example, certain sections of the 1992 Landers, California, earthquake rupture resulted in “100% off-fault deformation^19^,” indicating to us that no discrete offset was observed at Earth’s surface and the primary rupture remained buried. Furthermore, a study of >1200 fault strands in paleoseismic trenches found that >70% of reverse and strike-slip fault strands could not be traced to the surface that existed at the time of faulting^75^. Previous work has suggested that nonlinear interaction of seismic waves can cause near-surface yielding ahead of a rupture, which may lead to arrest^76^. The similar deformation and inferred rupture depths at the Buhman and Saintsbury sites, however, demonstrate that shallow rupture arrest is not – at least not exclusively – a dynamic effect.

Our models assume that the distributed deformation observed at Earth’s surface during the South Napa event resulted from slip on a buried rupture, defined as a narrow structure across which the majority of shear displacement localizes. This inference is supported by geological studies of exhumed fault structures, which typically indicate that the majority of displacement occurs within a highly localized zone^25,77–85^. For example, studies of southern California’s Punchbowl fault, exhumed from 2–4 km depth, indicate that only 100 m of the total 10 km right-lateral displacement was accommodated within a 100 m wide damage zone; most of the displacement occurred within a ~30 cm thick ultracataclasite core, with at least 2 km displacement further localized within a ~2 cm thick principal slip zone^25,77^. Observations made closer to Earth’s surface and along active faults provide additional evidence for slip localization. For example, paleoseismic trenches along geometrically simple sections of historically-ruptured faults show that deformation at shallow (~3–8 m) depth often focuses within zones <1 cm wide, though fault structure often “flowers” upward toward Earth’s surface where the observed deformation may be distributed across a zone that is tens-of-meters wide^32,86–88^. Additionally, boreholes crossing actively creeping strike-slip faults record concentrated deformation within zones narrower than 3 m wide, even within alluvium and at <25 m depth^89,90^. Furthermore, deformation of subsurface infrastructure, including tunnels^91^ and qanats (aqueducts)^79^, during historic earthquakes indicates localized fault slip within tens-of-centimeters at shallow depth (~30 m), even where deformation was distributed across tens- to hundreds-of-meters at Earth’s surface.

In addition to geological observations, theoretical mechanics suggests that deformation should occur within a narrow zone at depth. At seismogenic depths where earthquake ruptures nucleate and propagate, thermally-driven dynamic weakening mechanisms require slip to localize within several centimeters^79^ to as narrow as <50 microns at 7 km depth^92,93^. Near Earth’s surface, continuum deformation governed by frictional plastic yielding (e.g., Drucker-Prager, Mohr-Coulomb) should progressively narrow with depth as mean normal stress increases^7,22^. Thus, we expect that the width of the deformation zone observed along the South Napa rupture should taper downward toward a much narrower zone of localized shearing. At the Saintsbury site, the observation of a 2–3 cm wide principal slip zone in drill core collected at 9 m depth supports this hypothesis^53^. It is not possible from the core to determine how much offset accrued across the localized clay-rich shear zone. Based on empirical displacement-thickness (D-T) scaling relations (linear with D/T ranging from 10 to 100 for brittle faults^94^), we infer that this 2–3 cm thick zone accrued significantly more displacement than the subsidiary shears, which typically have thickness on the order of a millimeter or smaller^53^. Although we cannot absolutely eliminate the possibility that the deformation zone extends downward with constant width (i.e., the rupture has a finite width), this would be inconsistent with the mechanical theory and previous geological observations outlined above.

Our multi-disciplinary approach reveals several important sensitivities of shallow fault slip and near-field deformation. Mechanical models (Fig. 3) demonstrate that the effect of a compliant zone on shallow faulting is secondary to that of incorporating realistic lithologic layering and/or elastoplastic mechanical properties. This is consistent with our conclusions from the combined seismic and MLS data (Fig. 2), that the compliant zone at Saintsbury did not strictly control the observed shear strain distribution. Our observations are in contrast to previous studies documenting enhanced deformation confined to compliant zones^21,28^. Importantly, these previous studies evaluated triggered deformation within a compliant zone due to slip on a fault located >10 km away, whereas our study considers deformation due to slip on a fault located within the targeted compliant zone.

In contrast to compliant zones, we find that horizontally layered elastic stiffness representative of lithologic layering can significantly affect shallow fault behavior. Compared to a homogeneous elastic model, those including a soft surface unit overlying a stiff basement unit (Fig. 3c, “2. Layered E”) produce greater shallow slip and a more abrupt slip reduction at the fault tip. This occurs because, for the uniform driving slip below 50 m depth (see Supplementary Fig. 8), the stiffer basement material produces a greater shear stress perturbation. This leads to greater slip along the fault segment shallower than 50 m, which is solved for using the Coulomb criterion. Slip is further amplified if the surface unit is very soft, because greater shear strain can accumulate around the buried fault tip and resistance to slip is reduced (Supplementary Fig. 12). Because soft, unconsolidated material (e.g., alluvium) often overlies bedrock, slip may commonly decrease abruptly at buried fault tips. Although this has not been observed directly, recent studies of surface deformation along earthquake ruptures have documented primary slip surfaces buried in alluvium^95^ and systematic differences between near- and far-field displacements^16,19,56^, indicating a slip reduction within the shallowest tens to hundreds of meters of Earth’s crust.

Compared to the layered elastic models, elastoplastic models produce even greater shallow fault slip and more abrupt slip reduction at the buried fault tip (Fig. 3). This result is consistent with previous field observations of exhumed faults, where off-fault inelastic strain is associated with steep slip gradients (e.g., slip decreases from 10 cm to 0 cm over a distance of 10 cm)^73,96,97^, and with mechanical modeling of faults in Von Mises elastoplastic^73^ and non-linear viscous^98^ media. Whereas the elastic models produce slip gradients that would require stress near the fault tip to exceed the expected yield strength for near-surface materials, the elastoplastic models inherently limit the stress state to geologically appropriate values regulated by the Mohr-Coulomb yield criterion. Additionally, the elastoplastic models are consistent with independent evidence for plastic deformation during the South Napa event (i.e., mapped shear strain in Fig. 1, shear fabrics in trenches^12^, lower-than-expected ground motions^57,58^). Although we cannot use seismic data to constrain plastic properties, future work may allow us to infer qualitative variations in yield strength based on observations of plastic deformation features (e.g., penetrative fabrics evident from seismic anisotropy, “flower structure^99^” indicated by a reduction of shear wave velocity) and knowledge of whether the material is strain-hardening or -softening from mechanical testing of core samples.

Finally, our study encounters the long-recognized challenge of non-uniqueness inherent to all geodetic studies: surface displacement fields can be fit equally well by either elastic or elastoplastic models with different input parameters (Fig. 4a,b). The corresponding distributions of subsurface deformation, however, are distinct (Fig. 4c–h). Thus, without additional mechanical and/or structural constraints, we cannot rely on surface displacement data alone, regardless of the resolution, to develop meaningful mechanical models and to confidently infer shallow fault processes. This is problematic for scientists and engineers aiming to leverage the expanding field of near-field geodesy for various subsurface applications, for instance, utility companies maintaining buried pipelines, engineers designing structural foundations, and paleoseismologists constraining fault slip rates for hazard models. Distinguishing between possible subsurface scenarios from geodetic data (e.g., Fig. 4) has important ramifications, and requires that future analyses incorporate site-specific mechanical data to eliminate sources of non-uniqueness.

Where land access is not permitted, geodetic studies would benefit from additional knowledge of mechanical properties for a variety of geologic settings. Because geologic maps exist for most continental regions, particularly where active faults are recognized, constraining a site’s surface geology is nearly always possible. Then, a geodetically-constrained model can implement mechanical properties appropriate for that setting based on site-specific data acquisition and analysis possible at another locale with similar surface geology.

## Methods

### MLS data smoothing and shear strain calculation

Methods of MLS acquisition and processing are described elsewhere^12,100,101^. MLS point clouds for each vine row have significant scatter due to the presence of branches and leaves (Fig. 2a,b), making it necessary to smooth the data before further analysis. At Buhman, we analyze MLS data for 69 vine rows from the vineyard immediately adjacent to the seismic survey (Fig. 1b). Due to suboptimal orientation of vine rows at Saintsbury, we analyze 83 vine rows from two vineyards that straddle the seismic survey at distances of 200–300 m (Fig. 1c). We smooth the data using the non-parametric technique of Nadaraya-Watson Gaussian kernel regression^102,103^. For each vine row, we use a bandwidth of 3.5 m and 500 regression points, which provides a visibly smooth approximation and captures the prominent deflections along each row (Fig. 2a,b).

We use the smoothed curves to calculate shear strain assuming offset along each vine row resulted from fault slip during the South Napa earthquake. We first rotate each vine row such that the mapped fault trace parallels the y-axis. We then use the linear regression function in MATLAB to fit the limbs (i.e., the straight segments of each vine row not deformed during the South Napa earthquake) at ±15 m from the mapped fault trace. We average the slopes for the two limbs, and assume the undeformed (i.e., pre-earthquake) vine row followed this average trend through the fault zone. We then calculate shear strain in 1 m increments as: *ε*_*xy*_ = 0.5 d*u*_*y*_/d*x*. with the y-coordinate aligned with the fault trace and the x-coordinate extending orthogonally in the horizontal plane. We plot shear strain profiles together (Fig. 2c,d) by setting the x-coordinate corresponding to maximum shear strain to zero for all profiles, effectively centering each profile at the primary fault trace. For both the Buhman and Saintsbury sites, we then compute average shear strain distributions within 1 m increments (Fig. 2c,d).

### Seismic data acquisition and processing

At each study site, we conduct active-source seismic surveys along a ~120 m long transect approximately perpendicular to the fault trend (Fig. 1b,c). The survey includes 118 geophones at Buhman and 116 geophones at Saintsbury. P- and S-wave data are collected in sequence using the same station geometry with 1 m spacing. Seismic sources are generated within ~30 cm of each geophone. P-wave sources are produced by a 4.5 kg hammer vertically striking a steel plate on the ground surface, while S-wave sources are produced by a 4.5 kg hammer horizontally striking an aluminum block that is tethered to the ground surface. Both P- and S-wave data are recorded on two 60-channel Geometrics Stratavisor seismographs connected to 40 Hz Sercel vertical-component geophones and 4.5 Hz Sercel horizontal-component geophones, respectively. The horizontal geophones are oriented perpendicularly to the transect.

We locate geophones to better than ~1 cm accuracy in a global reference frame by surveying each station with dual frequency GPS equipment. For each line, a continuously operating base station is operated within 50 meters of the seismic stations. Each geophone location is surveyed by a rover GPS unit for more than a minute at 1 Hz sampling frequency. The ensemble of the base station and rovers are processed using kinematic carrier phase processing software (GrafNav) and placed in a global reference frame using a nearby continuous GPS base station.

We generate seismic tomography models based on first arrival travel times and a previously published modeling code^104^. Because geophone and shot locations are collocated with 1 m spacing, and due to the redundancy of having a shotpoint at every geophone, we parameterize the P- and S-wave velocity models using 1 m horizontal (x) and vertical (z) intervals. For the tomographic inversions, we use 1D starting models developed from shot-gather modeling. Significant smoothing in both x- and z-directions is used for initial inversion iterations, with the amount of smoothing slowly decreasing with iteration number. We expect high (<5 m) spatial resolution at both sites, given the high spatial density (1 m spacing) of the sources and receivers, which resulted in >10,000 first break arrival picks to invert each model (both P- and S-wave)^61,105^.

Elastic moduli (Fig. 2e–j) are determined from P-wave velocities (V_p_) and S-wave velocities (V_s_) inferred from the tomography model assuming uniform density, *ρ* = 2000 kg/m^3 ^^71^, and an isotropic linear elastic solid with the following equations: *G* = *ρ*V_s_^2^; *ν* = [V_p_^2^ − 2V_s_^2^]/[2 (V_p_^2^ − V_s_^2^)]; *E* = 2*G* (1 + *ν*)^106^.

### Finite element model

Finite element models are constructed and analyzed using Abaqus FEA^107^ with the Standard implicit solver accounting for geometric nonlinearity. To reduce computation time, an iterative solver based on the Krylov method^108^ is used with the default convergence tolerance (10^−6^ and 10^−3^ for linear and nonlinear perturbations, respectively). All models presented here converged without error.

The 3D model domain (Supplementary Fig. 8) measures 2.5 km by 2.5 km by 1 km in the x- y- and z-directions, respectively. We develop the model in three dimensions so that future work building upon this study can consider fault tips with irregular geometries (e.g., the depth of the fault varies along strike). A vertical, planar fault bisects the model, with the fault surfaces partitioned at 2 m, 5 m, 10 m, and 50 m depths to allow for variations in boundary conditions in the models. Contact properties for the fault prevent interpenetration between the two sides of the fault. In the base model (i.e., Fig. 3), fault surfaces above 5 m depth are tied (i.e., forbidden to slip), such that the fault tip is effectively buried to that depth. An additional volume partition is defined at x = +/−25 m from the fault surface, to allow the definition of a compliant zone, and at z = −10 m to allow for layered heterogeneities (i.e., surface versus basement units). Models use >260,000 quadratic (10-node) tetrahedral elements with sizes ranging from 1 m at the fault to 150 m at the model boundary. We require the mesh to pass the Abaqus verification test with default values, ensuring that each element has a shape factor (element volume/volume of equilateral tetrahedron) >0.0001, face corners >5° and <170°, and an aspect ratio (longest element edge/shortest element edge) <10. Models assume isotropic, linear elasticity or elastoplastic behavior following Mohr-Coulomb yielding with perfect plasticity and non-associative flow (Table 1)^109^. Previous studies have shown near-surface granular materials to follow frictional plastic yield criteria, including Mohr-Coulomb, with non-associated flow^69^. We use the Mohr-Coulomb, rather than Drucker-Prager, yield criterion in order to minimize assumptions (e.g., yield envelope convexity, whether the yield envelope is inscribed or circumscribed) and approximations that are otherwise required to match laboratory-constrained parameters (i.e., cohesion and angle of internal friction) to the Drucker-Prager model, particularly when the angle of internal friction exceeds 22°^107^. All models assume homogeneous density *ρ* = 2000 kg/m^3^ and fault friction *µ* = 0.4.

Boundary conditions are introduced in two steps: (1) Gravitational loading to instate a lithostatic pressure gradient. The gravitational load is in equilibrium with an initial geostatic stress field defined for the model domain. By the end of the first step, the gravitational load generates <2E-5 m vertical displacement and <4 Pa Mises equivalent stress across the model, along with the desired lithostatic pressure gradient, which increases linearly from 0 MPa at the modeled Earth’s surface to 19.6 MPa at the base of the model; (2) Prescribed uniform right-lateral fault slip of 0.5 m below 50 m depth. Because the MLS data do not consistently capture surface deformation beyond ±50 m from the fault, our comparison between model results and MLS data is restricted to that very near-field region. Our models assume uniform slip below 50 m, though fault slip during the South Napa earthquake likely was more complicated. We evaluate our assumption of uniform driving slip using a series of model tests (Supplementary Fig. 9, Supplementary Table 1), which demonstrate that reasonable distributions of heterogeneous driving slip below 50 m depth would not significantly affect the modeled surface deformation within the ±50 m near-field region of consideration. No other pre-stress is applied to the model domain, whereas the shallow crust may have been close to failure when the earthquake occurred. Thus, the models may underestimate the magnitude and spatial distribution of plastic strain.

The bottom model boundary and each vertical fault-parallel boundary have zero displacement defined for the boundary-orthogonal direction during both steps (Supplementary Fig. 8). The vertical boundaries orthogonal to the fault are subjected to lithostatic pressure gradients in both steps, allowing the fault to slip all the way to these boundaries without contradictory displacement boundary conditions. We benchmarked this model using the Okada (1985) dislocation solution^110^ for slip on a vertical planar fault embedded in a homogeneous elastic medium, and using the Du *et al*. (1994) dislocation solution^40^ for slip on a vertical planar fault embedded in a layered elastic medium (Supplementary Fig. 13).

Output was recorded on two node sets: (1) a transect at the modeled Earth’s surface orthogonal to the fault and across the entire model domain, from [*x*, *y*, *z*] = [−1.25 km, 0 km, 0 km] to [1.25 km, 0 km, 0 km]; (2) a vertical array from the surface to 50 m depth at the center of the fault, from [*x*, *y*, *z*] = [0 m, 0 m, 0 m] to [0 m, 0 m, −50 m]. Surface displacements (Figs. 3a,b, 4a,b) and fault slip (Figs. 3c,d, 4c,d) were recorded along these node sets, respectively.

## Supplementary information

Supplementary Information.

## Data Availability

Datasets generated for and analyzed during this study are available as U.S. Geological Survey data releases^113,114^ and from the corresponding author upon reasonable request.

## Code availability

Abaqus input files and MATLAB scripts supporting the findings of this study are available from the corresponding author upon reasonable request.
