# Supplementary material for: Mechanics of near-field deformation during co- and post-seismic shallow fault slip
Source: Sci Rep. 2020 Mar 19;10:5031. doi: 10.1038/s41598-020-61400-9 (PMC7081326; doi:10.1038/s41598-020-61400-9)
Supplement: Supplementary file 1 — Supplementary Information. [file 41598_2020_61400_MOESM1_ESM.pdf]

Supplementary Information for

Mechanics of near-field deformation during co- and post-seismic shallow fault slip

Johanna M. Nevitt[1]*, Benjamin A. Brooks[1], Rufus D. Catchings[1], Mark R. Goldman[1], Todd L. Ericksen[1], Craig L. Glennie[2]

[1] U.S. Geological Survey, P.O. Box 158, Moffett Field, CA 94035 USA

[2] Department of Civil and Environmental Engineering, University of Houston, Houston, TX 77204 USA

*Corresponding author: jnevitt@usgs.gov

Contents:

- Supplementary Notes 1-2
- Supplementary Figures 1-13
- Supplementary Table 1
- References

Supplementary Note 1: Uncertainty in tomographic models of elastic moduli

Uncertainty in the elastic moduli is due primarily to the assumption of uniform density. We evaluate this assumption by re-calculating the tomographic models using variable density according to Gardner's Relation, an empirical equation relating P-wave velocity, V_p, in [m/s] to density, ρ in [g/cm^3]: $\rho = 0.31 V_p^{0.25}$ [1]. Using Gardner's Relation, we find that at Buhman, density varies from ~1400-2400 kg/m^3, with density increasing from the surface toward the bottom of the model. At Saintsbury, we find that density varies from ~1400-2000 kg/m^3, again with density increasing from the surface toward the base of the model (Supplementary Figure 3).

Using the variable density models to re-compute the tomographic models (Supplementary Figures 4-6) does not lead to significant changes for the Buhman study site, with the overall shape of the distributions looking very similar to the uniform density model. Results indicate that the uniform density model may overestimate G (by <~30 MPa) and E (by <~80 MPa) in the shallow portions of the model, and underestimate G (by <60 MPa) and E (by <~170 MPa) in the deepest portions of the model. Thus, the contrast between soft near-surface lithologic layer and the stiffer basement unit may be slightly more pronounced than the values used in the finite element models (based on uniform density). Based on model results presented in this manuscript, we expect a more pronounced stiffness contrast between lithologic layers to result in greater slip below 10 m depth.

At Saintsbury, using variable density from Gardner's Relation does alter the shape of the elastic moduli distributions (Supplementary Figures 4-6). The variable density model leads to a reduction in the overall magnitudes of G (by <~40 MPa) and E (<~80 MPa), with the largest differences in the shallowest portion of the model and with increasing lateral distance from the fault. In addition, the concentric contours defining the low G and E zones surrounding the fault become broader and flatter compared to the uniform density model. This results in a wider compliant zone (~80 m wide for 50% reduction in G) compared to that calculated for the uniform density model. Supplementary Figure 6 indicates that the overall reduction in G and E would lead to less fault slip in a homogeneous model. We don't expect the broadening of the compliant zone to exert much influence, since the spatial distribution of the shear strain is not strictly controlled by the dimensions of the compliant zone.

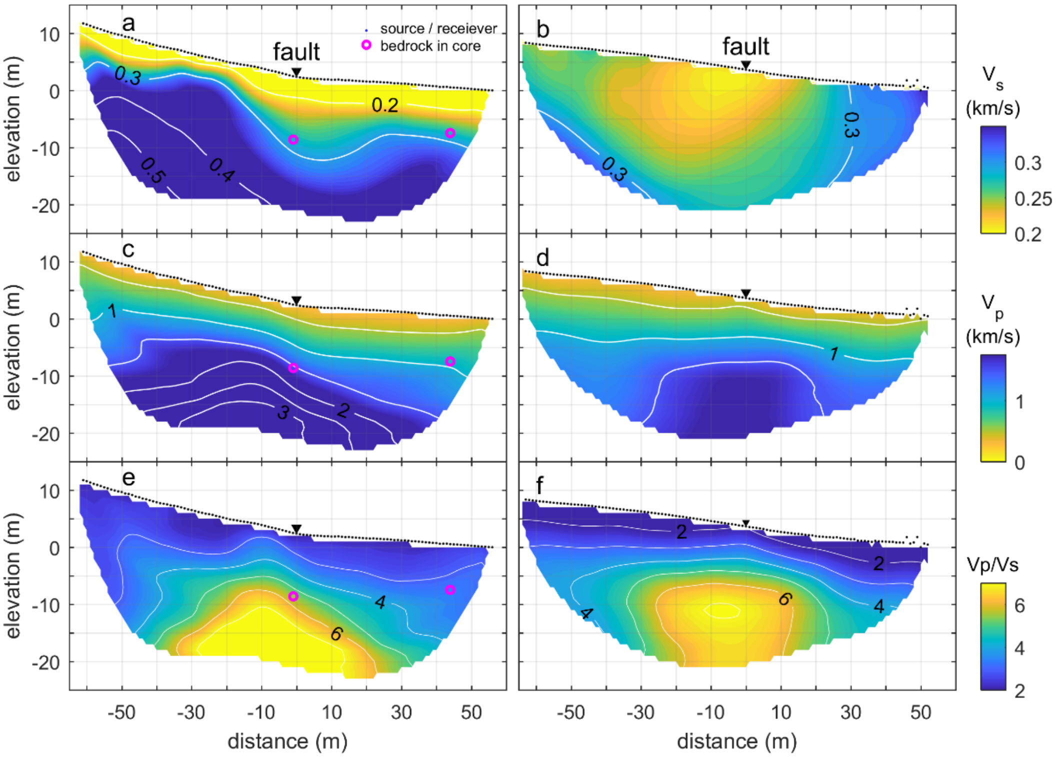

Supplementary Figure 1. Seismic velocities and their ratio for Buhman (left) and Saintsbury (right) study sites: (a-b) V_s; (c-d) V_p; and (e-f) V_p/V_s.

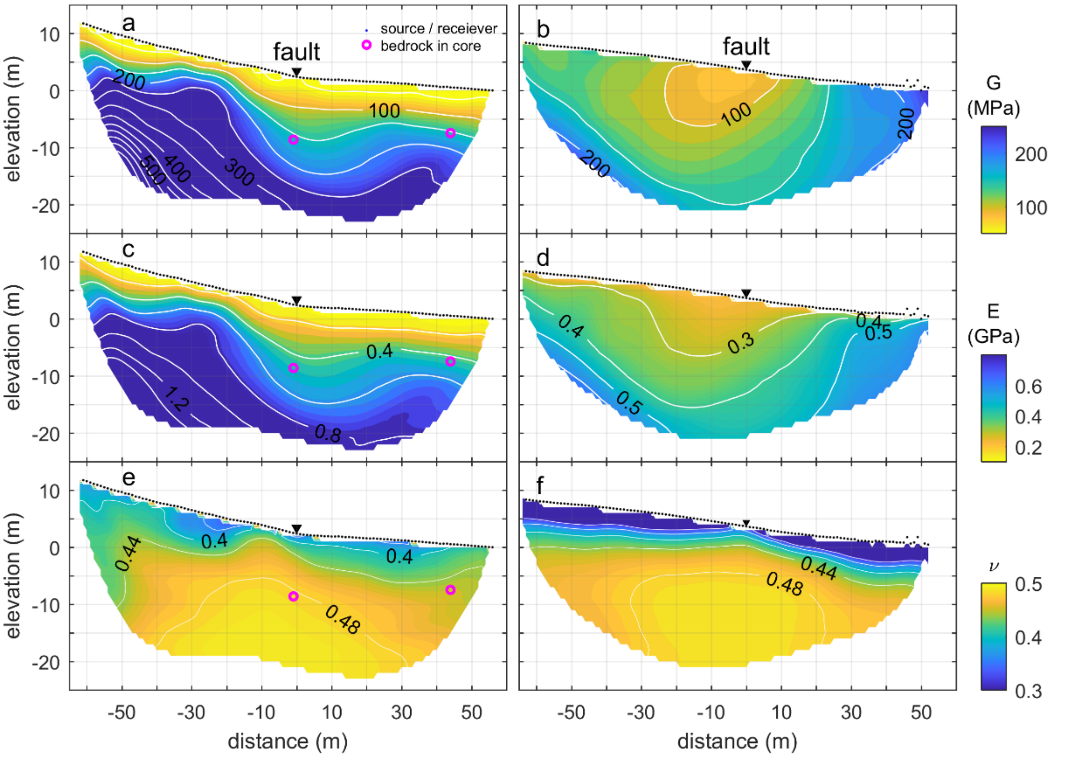

Supplementary Figure 2. Un-normalized elastic moduli for Buhman (left) and Saintsbury (right) study sites assuming uniform density (2000 kg/m^3) and a linear elastic isotropic solid: (a-b) Shear modulus, G; (c-d) Young's modulus, E; and (e-f) Poisson's ratio, ν. Equations for generating these plots are included in the Methods section.

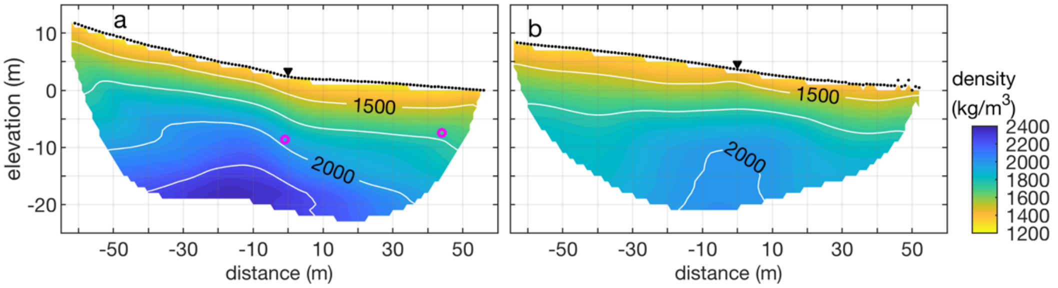

Supplementary Figure 3. Density models for the (a) Buhman and (b) Saintsbury study sites, calculated using Gardner's Relation, $\rho = 0.31\ V_p^{0.25}$ (Reference), an empirical equation relating V_p in [m/s] to ρ in [g/cm^3].

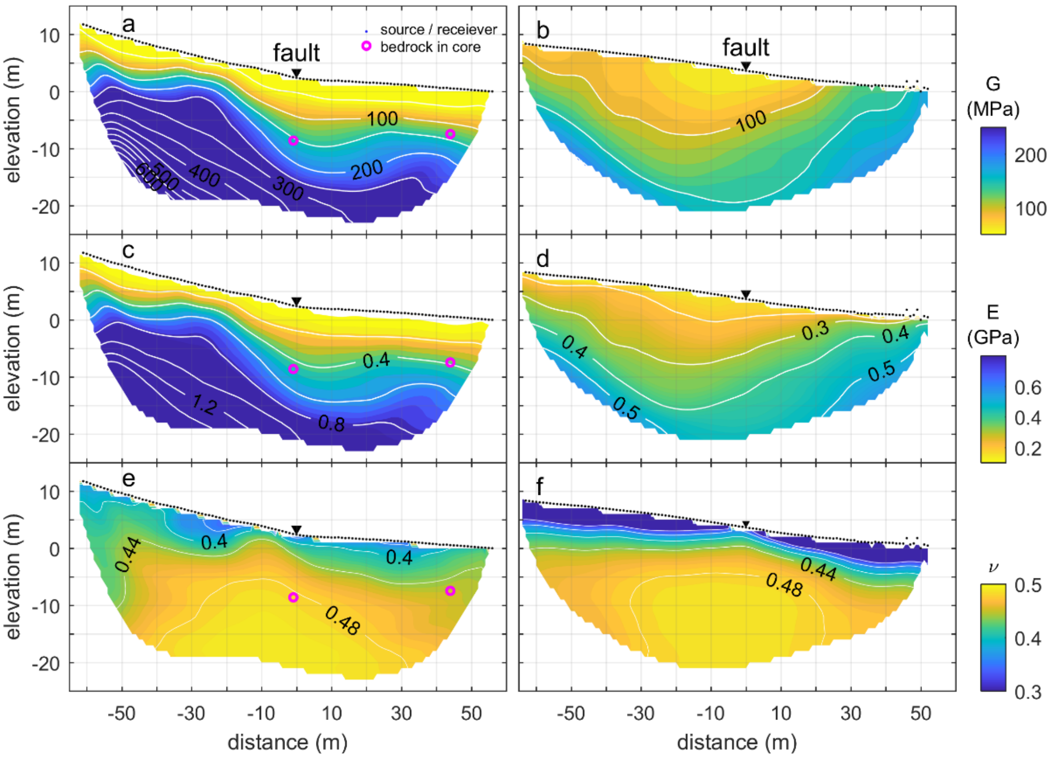

Supplementary Figure 4. Un-normalized elastic moduli for Buhman (left) and Saintsbury (right) study sites using variable density models from Gardner's Relation (Supplementary Figure 3) and assuming an isotropic linear elastic solid: (a-b) Shear modulus, G; (c-d) Young's modulus, E; and (e-f) Poisson's ratio, ν. Equations for generating these plots are included in the Methods section.

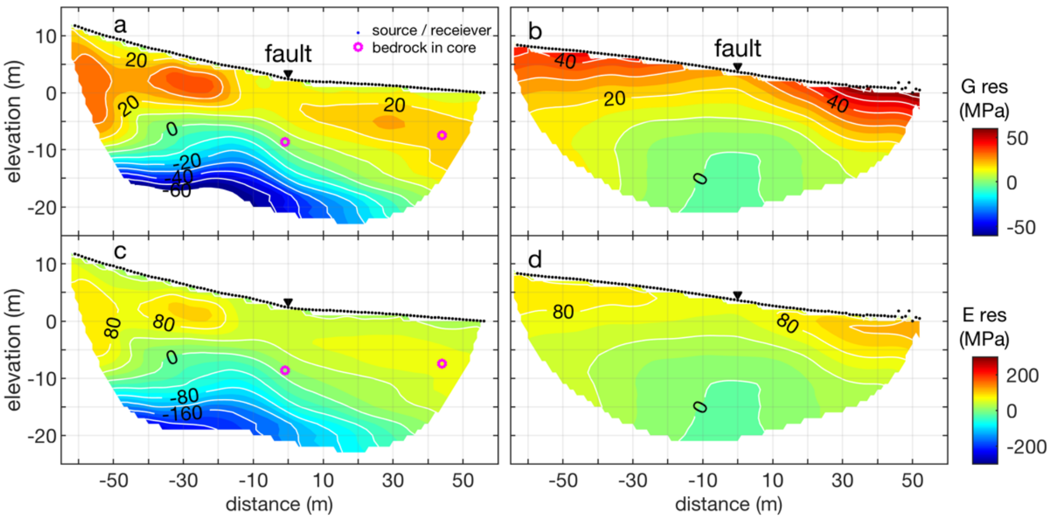

Supplementary Figure 5. Residual between tomographic models calculated using uniform density and variable density from Gardner's Relation for Buhman (left) and Saintsbury (right) study sites: (a-b) G res = G ($\rho_{uniform}$) – G ($\rho_{Gardners}$); (c-d)) E res = E ($\rho_{uniform}$) – E ($\rho_{Gardners}$). Note that Poisson's ratio is not a function of density.

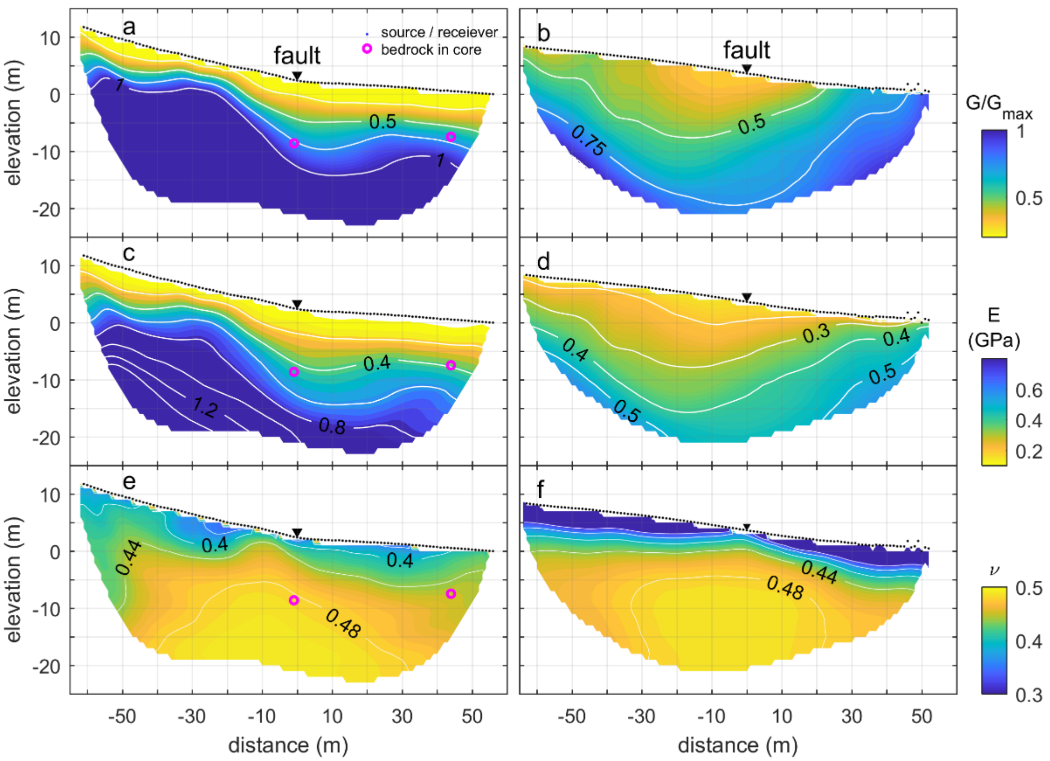

Supplementary Figure 6. Elastic moduli for Buhman (left) and Saintsbury (right) study sites using variable density models from Gardner's Relation and assuming an isotropic linear elastic solid: (a-b) Shear modulus normalized by approximately the maximum value at the Saintsbury site, 200 MPa, G/G_{max}; (c-d) Young's modulus, E; and (e-f) Poisson's ratio, ν. Equations for generating these plots are included in the Methods section.

Supplementary Note 2: Observations from fault zone drilling

Using a hollow-stem auger drill rig, we collected two continuous core samples at the Buhman study site (Figure 1b) in stainless steel 3-inch-diameter shelby tubes. The first borehole is located in the center of the fault zone and reached a depth of 38'11.5" (~12 m). Recovered material from shallow depths consists largely of brown-grey sandy- to silty-clay (Supplementary Figure 1a) with occasional gravel lenses. At ~35 ft (~11 m) depth, augering became very difficult, with the rig lifting off its support pads. The exposed sample at the base of the recovered shelby tubes and within the auger was sandstone (Figure 2, Supplementary Figure 7b). This is consistent with field mapping at the site [2] that indicates the presence Great Valley Group sandstone. We also observe sandstone outcropping in a creek bed ~80 m NNW of the borehole. At the second borehole, located 50 m ENE downslope of the center of the fault zone (Figure 1b), we encountered similar materials with sandstone located at 25' (~8 m) depth.

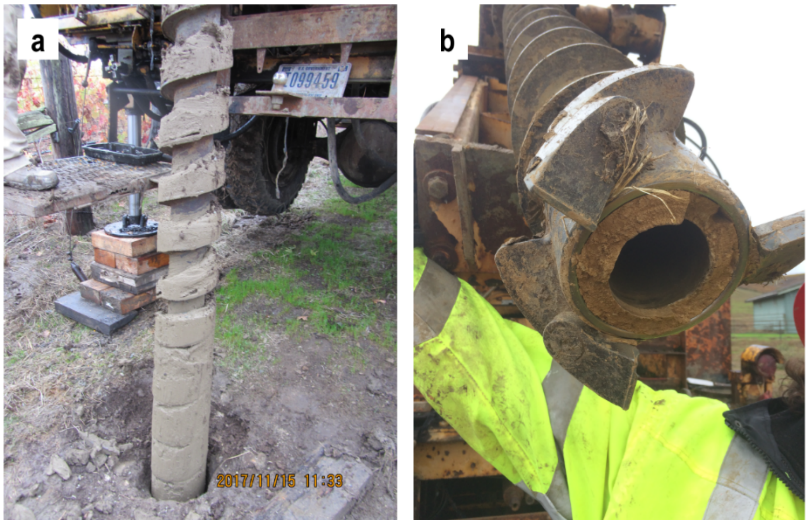

Supplementary Figure 7. Observations made while drilling the fault zone borehole the Buhman study site (location shown in Figure 1b). (a) At depths above ~11 m, the auger primarily returned coated in cohesive brown-grey silty- to sandy-clay; (b) The auger returned from ~12 m depth clean and packed with sandstone, indicated a change in lithology at ~11-12 m depth.

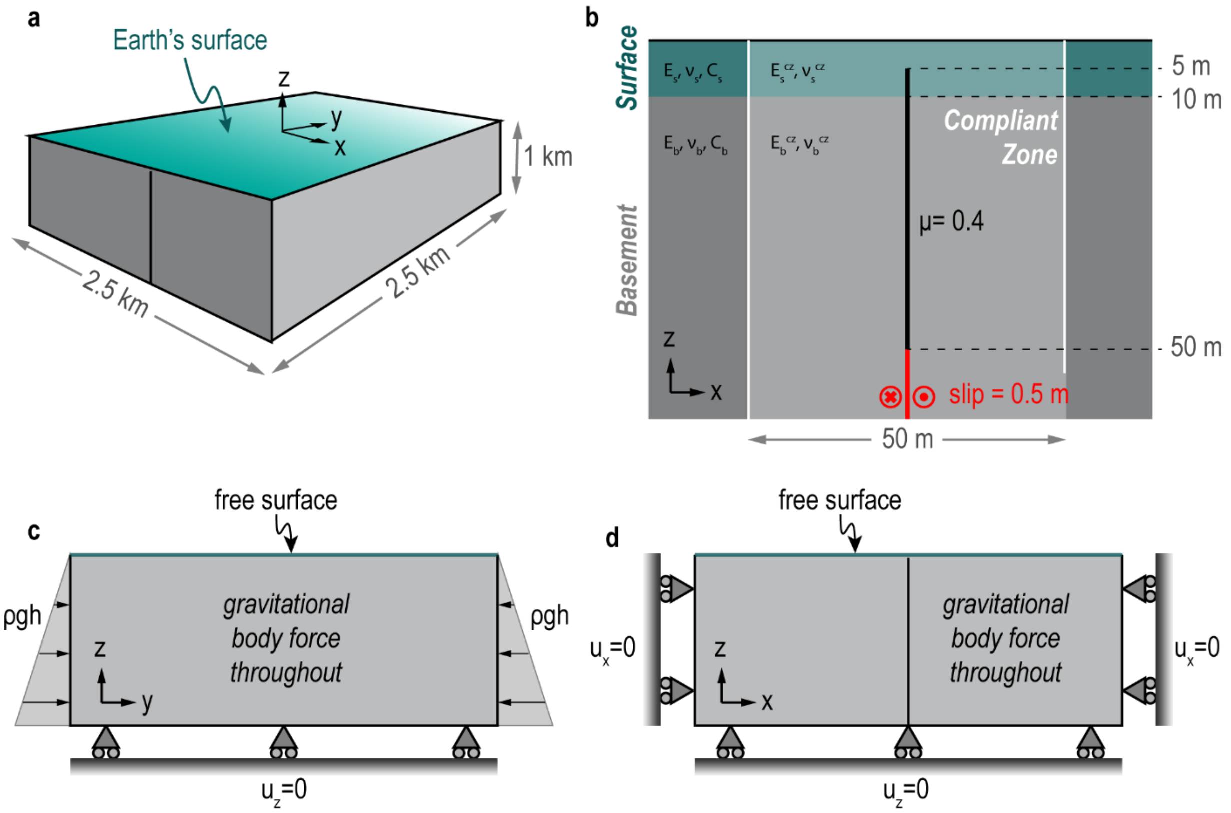

Supplementary Figure 8. Geometry and boundary conditions used in the finite element models: (a) Dimensions of full model domain; (b) Cross-sectional inset perpendicular to the fault in the center of the model. The model has a 10 m deep horizontal partition to allow distinction between mechanical properties near Earth's surface and deeper, basement units. Similarly, there is a vertical volumetric partition at x = ±25 m, to allow for a compliant zone. In the base model (Figure 3), slip of 0.5 m is prescribed below 50 m depth, but its value is calculated above that depth according to the Coulomb criterion with coefficient of friction, $\mu = 0.4$. For model results presented in Figure 4 only, we tailored the prescribed slip to fit the vine row data: 0.55 m for Buhman, 0.45 m for Saintsbury. Subscripts "b" and "s" refer to basement and surface units, respectively, while superscript "cz" indicates the compliant zone. Properties varied in the models include Young's modulus (E), Poisson's ratio (ν), and cohesion (C) (see Table 1); (c-d) Boundary and initial conditions shown for two orthogonal cross-sections through the model, where ρgh is the lithostatic load applied to the fault-orthogonal boundaries and u_i are displacement components.

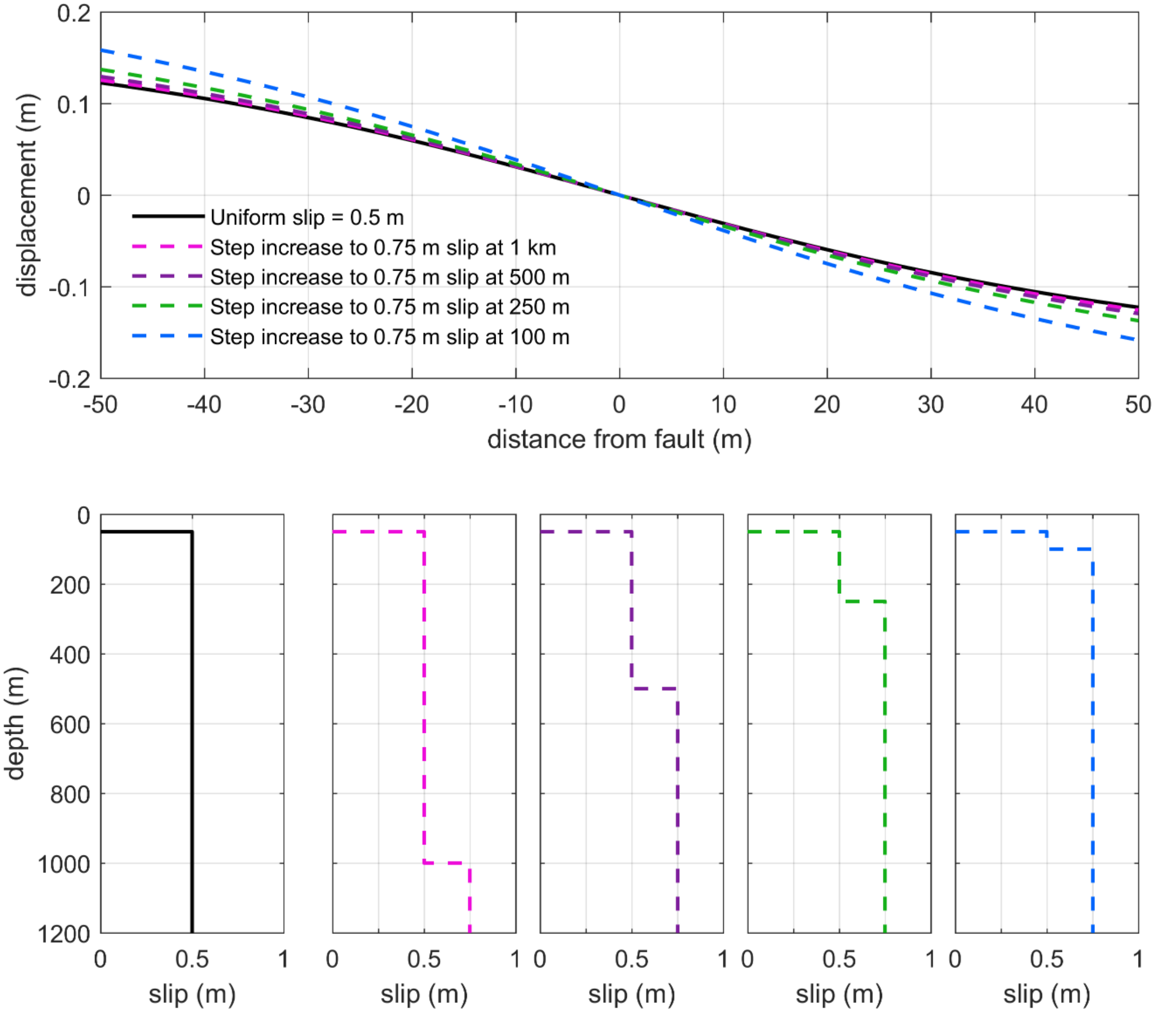

Supplementary Figure 9. Variation in near-field surface deformation (top panel) due to heterogeneous driving slip below 50 m depth (bottom panels). Kinematic and static inversions of surface deformation from the 2014 South Napa earthquake suggest that both co-seismic and post-seismic slip at depths shallower than 1 km was less than ~0.75 m [3-7]. Here we present Okada (1985) analytical solutions for a vertical finite rectangular source [8] with variable slip distributions. The source is 12 km long and 10 km deep, consistent with estimates for the South Napa earthquake rupture. In addition, the source has slip prescribed only to 50 m depth, which allows us to evaluate the assumption in the finite element models (Figures 3-4) that heterogeneous driving slip below 50 m would not significantly affect our comparison between model results and MLS data within 50 m of the fault. We compare the solutions of four models where 0.75 m driving slip reaches depths of 1 km (pink), 500 m (purple), 250 m (green), and 100 m (blue) depth with the solution for a model with uniform driving slip of 0.5 m (black, and the driving slip for the finite element models in Figures 3-4, Supplementary Figure 8). Within ±50 m of the fault, there is little deviation in the surface displacements between the models (see Supplementary Table 1). The model with a step increase at 100 m depth (blue) produces the greatest misfit from the uniform slip model (black) by an average amount (1.87±1.05 cm) near the resolution threshold of MLS.

Supplementary Table 1. Quantification of the global misfit between models with uniform slip versus variable slip below 50 m depth (see Supplementary Figure 9)

Depth where slip increases from 0.5 m to 0.75 m	Mean (cm)	Standard Deviation (cm)	Maximum (cm)
**1 km**	0.15	0.09	0.29
**500 m**	0.34	0.20	0.68
**250 m**	0.75	0.43	1.45
**100 m**	1.87	1.05	3.57

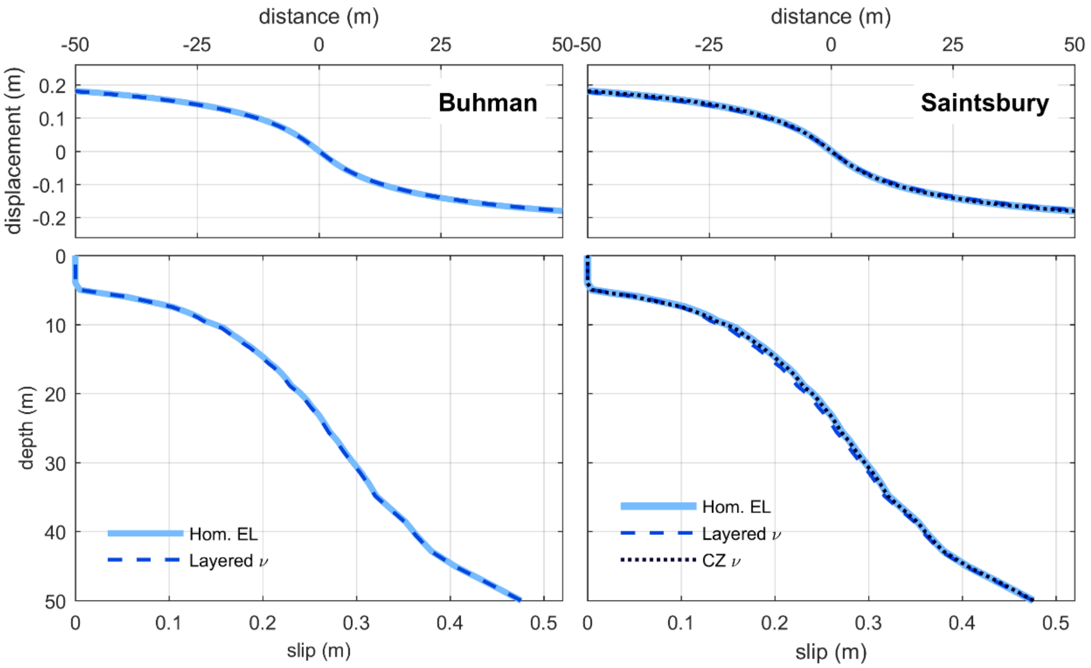

Supplementary Figure 10. Varying Poisson's ratio (ν) has little effect on fault slip (bottom) and surface deformation (top) for both study sites. "Hom. EL" is the homogeneous elastic base model, with $\nu = 0.44$ (see Table 1 for full description of properties used). At Buhman, the "Layered ν" model has $\nu_s = 0.40$ and $\nu_b = 0.44$. At Saintsbury, the "Layered ν" model has $\nu_s = 0.30$ and $\nu_b = 0.44$. The "CZ ν" defines ν within the compliant zone as 0.48; outside the compliant zone $\nu = 0.44$. All models shown are elastic with values based on seismic tomography models (Figure 2i-j).

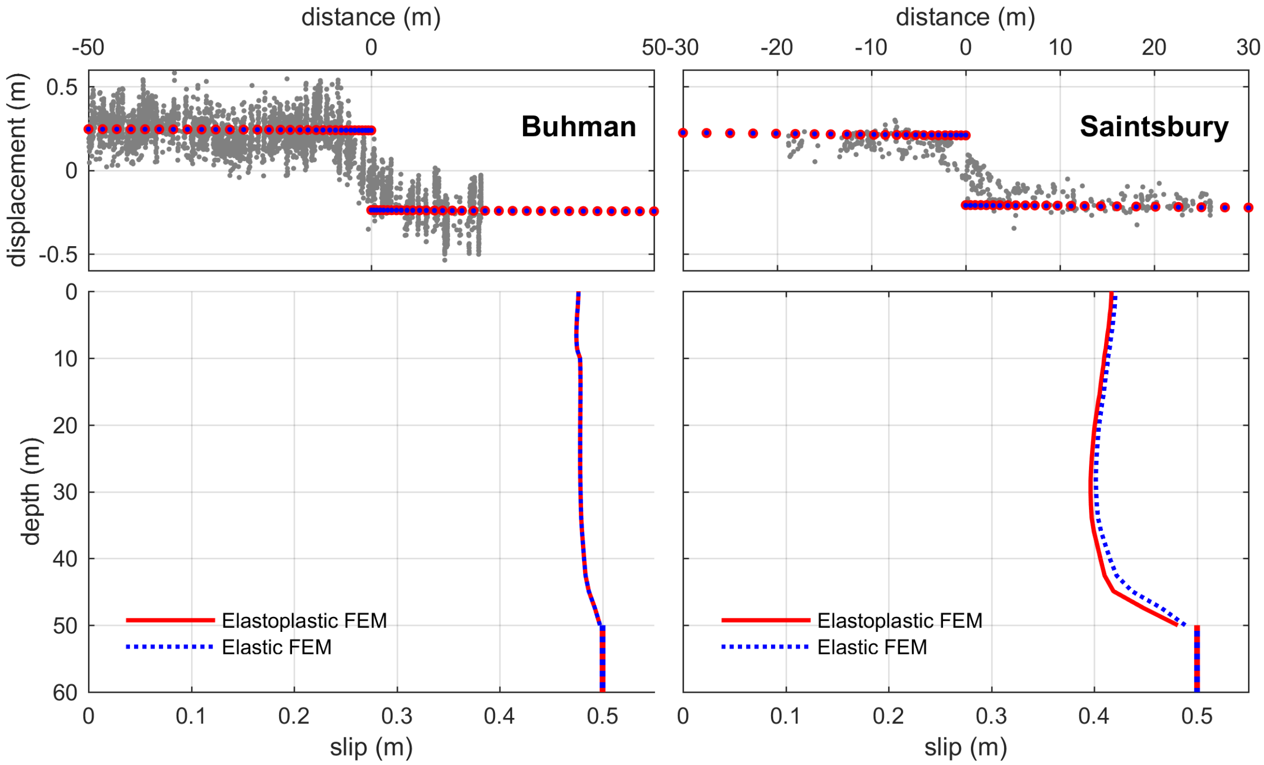

Supplementary Figure 11. Models that allow the fault to reach the free surface provide a poor match to surface displacement data from mobile laser scanning (grey circles in upper plots). Mechanical properties used in elastic and elastoplastic models are the same as in Figure 4. Slip of 0.5 m is prescribed below 50 m depth, while shallower slip follows the Coulomb criterion. Though slip decreases above 50 m depth, significant slip (>40 cm) does reach the surface, which was not observed in the field following the 2014 South Napa earthquake.

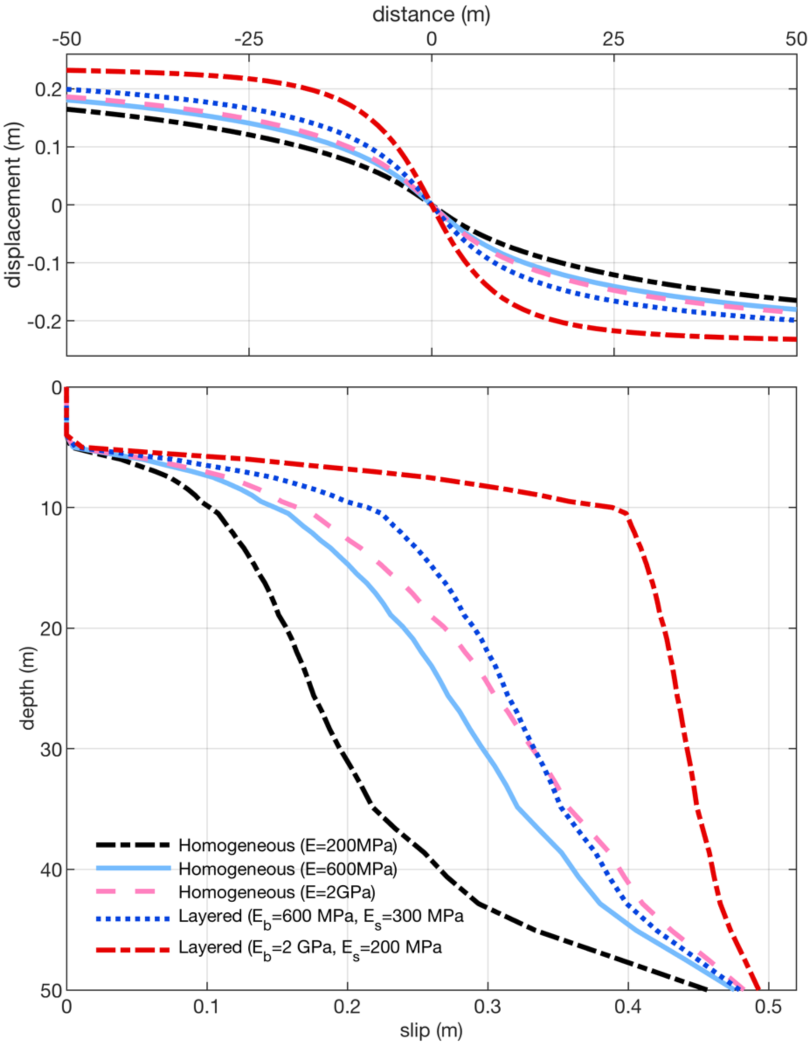

Supplementary Figure 12. The effect of model stiffness on surface displacements (top) and shallow fault slip (bottom) for homogeneous and layered elastic models. For a homogeneous model, increasing Young's modulus (E) results in greater fault slip for the section of the fault shallower than 50 m depth. This is because, for the uniform driving slip of 0.5 m prescribed along the fault below 50 m depth (Supplementary Figure 8), a stiffer continuum leads to a greater shear stress perturbation. As a result, fault slip shallower than 50 m depth, which is solved for using the Coulomb criterion, is greater than for models with a softer continuum. Introducing a soft surface unit above the stiff basement unit in the layered models amplifies the slip and leads to a steeper slip gradient at the fault tip, because greater shear strain can occur around a fault tip buried in a soft surface material compared to a stiff one.

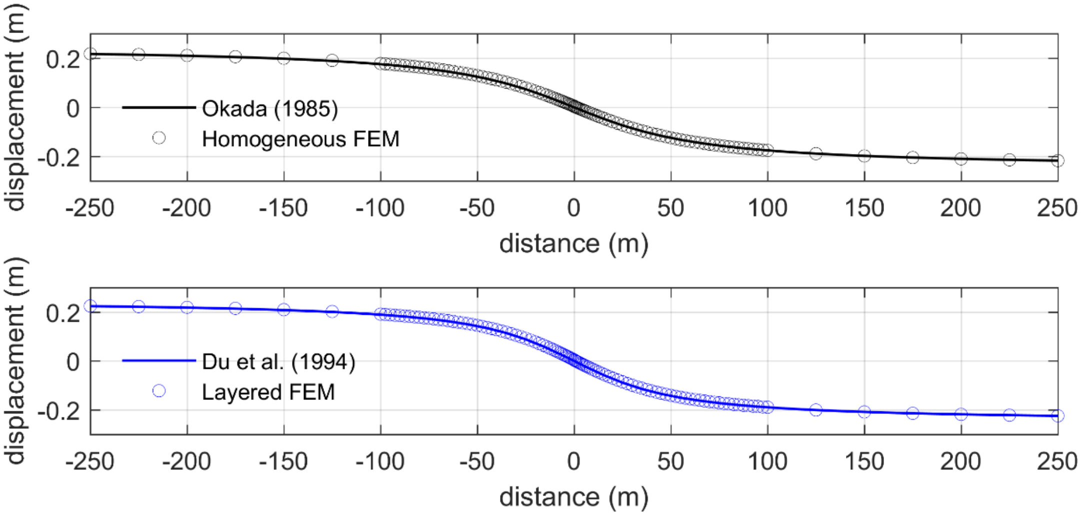

Supplementary Figure 13. We benchmarked the finite element model (FEM) using published solutions for a fault embedded in a homogeneous medium (Okada, 1985 [8]), and layered media (Du et al., 1994 [9]). In both models, the fault tip is buried 50 m with 0.5 m prescribed slip. For the layered models, the surface unit is 10 m thick with E = 100 MPa. The basement unit has E = 2 GPa. Maximum misfit between FEMs and published solutions is 0.5 cm, below the resolution threshold for mobile laser scanning. The homogeneous model has a global average misfit of 0.1±0.2 cm, while the layered model has a global average misfit of 0.2±0.1 cm.

References

1. Gardner, G., Gardner, L. & Gregory, A. Formation velocity and density—The diagnostic basics for stratigraphic traps. *Geophysics* **39**, 770-780 (1974).
2. Graymer, R. W. *et al.* Geologic Map of the San Francisco Bay Region. *USGS Scientific Investigations Map* (2006).
3. Ji, C., Archuleta, R. J. & Twardzik, C. Rupture history of 2014 Mw 6.0 South Napa earthquake inferred from near-fault strong motion data and its impact to the practice of ground strong motion prediction. *Geophys Res Lett* **42**, 2149-2156 (2015).
4. Wei, S. *et al.* The 2014 Mw 6.1 South Napa earthquake: A unilateral rupture with shallow asperity and rapid afterslip. *Seismological Research Letters* **86**, 344-354 (2015).
5. Melgar, D. *et al.* Seismogeodesy of the 2014 Mw6. 1 Napa earthquake, California: Rapid response and modeling of fast rupture on a dipping strike-slip fault. *Journal of Geophysical Research: Solid Earth* **120**, 5013-5033 (2015).
6. Barnhart, W. D. *et al.* Geodetic constraints on the 2014 M 6.0 South Napa earthquake. *Seismological Research Letters* **86**, 335-343 (2015).
7. Dreger, D. S., Huang, M. H., Rodgers, A., Taira, T. a. & Wooddell, K. Kinematic finite-source model for the 24 August 2014 South Napa, California, earthquake from joint inversion of seismic, GPS, and InSAR data. *Seismological Research Letters* **86**, 327-334 (2015).
8. Okada, Y. Surface deformation due to shear and tensile faults in a half-space. *Bulletin of the Seismological Society of America* **75**, 1135-1154 (1985).
9. Du, Y., Segall, P. & Gao, H. Dislocations in inhomogeneous media via a moduli perturbation approach: General formulation and two-dimensional solutions. *Journal of Geophysical Research: Solid Earth* **99**, 13767-13779 (1994).
